# Anaesthetics-Induced Neurotoxicity in Developing Brain: An Update on Preclinical Evidence

**DOI:** 10.3390/brainsci4010136

**Published:** 2014-03-14

**Authors:** Zhaowei Zhou, Daqing Ma

**Affiliations:** 1 Section of Anaesthetics, Pain Medicine & Intensive Care, Department of Surgery & Cancer, Faculty of Medicine, Imperial College London, Chelsea and Westminster Hospital, London SW10 9NH, UK; E-Mail: zzw0701@msn.com; 2 Department of Surgery, Royal Bolton Hospital, Minerva Road, Farnworth, Bolton, Lancashire BL4 0JR, UK

**Keywords:** anaesthetics, neuroapoptosis, neurotoxicity, neonates, developing brain

## Abstract

Every year millions of young people are treated with anaesthetic agents for surgery and sedation in a seemingly safe manner. However, growing and convincing preclinical evidence in rodents and nonhuman primates, together with recent epidemiological observations, suggest that exposure to anaesthetics in common clinical use can be neurotoxic to the developing brain and lead to long-term neurological sequelae. These findings have seriously questioned the safe use of general anaesthetics in obstetric and paediatric patients. The mechanisms and human applicability of anaesthetic neurotoxicity and neuroprotection have remained under intense investigation over the past decade. Ongoing pre-clinical investigation may have significant impact on clinical practice in the near future. This review represents recent developments in this rapidly emerging field. The aim is to summarise recently available laboratory data, especially those being published after 2010, in the field of anaesthetics-induced neurotoxicity and its impact on cognitive function. In addition, we will discuss recent findings in mechanisms of early-life anaesthetics-induced neurotoxicity, the role of human stem cell-derived models in detecting such toxicity, and new potential alleviating strategies.

## 1. Introduction

Increasing experimental data in pre-clinical settings suggest that early exposure to commonly used general anaesthetics could cause widespread neuroapoptosis and long-term neurocognitive deficits. Recent epidemiological studies also showed that anaesthetic treatment in young children could possibly associate with potential neurocognitive impairement later in life [1,2,3,4,5]. This raised growing concerns about the use of general anaesthetics in obstetric and paediatric anaesthesia.

Many of the published reviews on pre-clinical evidence in the field of anaesthetics-induced neurotoxicity in young animals included laboratory findings from prior to the year 2010 [6,7,8,9,10,11]. The aim of this review is to summarise recently available laboratory evidence, especially those being published after 2010, in the field of anaesthetics-induced neurotoxicity and its impact on cognitive function. In addition, we will discuss new models used in investigating the effects of anaesthetics on immature neurons, recent findings in mechanisms of anaesthetics-induced neurotoxicity and new potential means to mitigate such effect.

## 2. New evidence on anaesthetics induced neuronal cell death and cognitive impairment.

Researchers in the past three years have continued to show that commonly used anaesthetic agents can cause widespread neuronal cell death in developing animal brains. There is a trend to move the focus of research towards more commonly used anaesthetic agents, such as sevoflurane and desflurane.

2.5% sevoflurane anaesthesia for 2 h at the day 14 of gestation in mice can lead to immediate increase of neuroapoptosis in fetal mouse brain tissues and may induce subsequent learning and memory impairment in offspring mice [12]. Moreover, 6-h exposures to equipotent doses of 7.4%–8.0% desflurane, 0.75%–2.0% isoflurane, or 1.1%–3.0% sevoflurane, all dramatically increased neocortical neuronal apoptotic cell death in postnatal days six to eight mice [13,14,15,16]. However, desflurane seems to induce the greatest levels of neuroapoptosis among equipotent doses of all three inhaled anaesthetics [16], whereas isoflurane causes more neuroapoptosis than equipotent doses of sevoflurane [13,16]. In adulthood, mice treated with 8% desflurane, 3% sevoflurane, or 2% isoflurane for 6 h as neonates all had impaired long-term memory. Desflurane, but not sevoflurane or isoflurane, could cause significant impairment in short-term spatial working memory [16]. In contrast, none of these anaesthetics were shown to cause abnormal emotional or anxiety-related behavioural responses in adult mice [16]. Mice treated with 6 h of 0.75% isoflurane or 1.1% sevoflurane as neonates did not have any long-term memory defects [13].

Repetitive 6-h exposures of 2.4% sevoflurane to female rats during gestation can significantly increase apoptosis of neurons in hippocampus of the offspring [17]. Similar neuroapoptotic effect was observed after a single 4-h exposure of 3%–5% sevoflurane [18] and a 6-h exposure of 2.3% sevoflurane to seven-day-old rat pups [19]. These studies also showed that sevoflurane exposure impaired progenitor cell proliferation and reduce neuronal nitric oxide synthase (nNOS) level [17,19]. In the hippocampus, nNOS is known to play an important role in learning and memory. In comparison to the findings in mice, single sevoflurane exposure in neonatal period can result in significant impairment of both short-term and long-term memory in adult rats [18,20,21]. However, persistent neuronal cell loss or behavioural retardation in rats at later age was not observed [19,21] and the impact of sevoflurane on short-term memory loss in rats seems to be inconsistent [20,22]. Moreover, the memory loss in adult rats can be potentially treatable with an intervention known as environmental enrichment. However, the mechanism by which environmental enrichment mitigates anaesthesia-related memory impairment remains to be determined [20,23].

Given the recent clinical finding that multiple exposures to anaesthesia and surgery before the age of two years increased the risk of cognitive impairment in children [2], Shen and colleagues designed an animal model in which postnatal, day-six mice were treated with 3% sevoflurane or 9% desflurane for 2 h daily for one or three consecutive days. They demonstrated that multiple exposures to sevoflurane in young mice induced cognitive impairment detected at around one month of age. It was associated with a significant increase in levels of pro-apoptotic inflammatory markers at the end of the three days sevoflurane anaesthesia. However, anaesthesia with 3% sevoflurane for 2 h once only and 9% desflurane for 2 h daily, for three days induced neither cognitive impairment nor neuroinflammation [23]. Using a similar animal model in which postnatal day seven rats were exposed to 2 h of 1.8% isoflurane once only, or once every the third day for three days, the data from another research group also showed that repeated exposure to isoflurane is associated with greater long-term memory impairment than single exposure [24]. This is consistent with Zhu and colleagues’ finding that adult rats and mice that received 35 minutes of 1.7% isoflurane daily, for five consecutive days, from postnatal day 14 were cognitively impaired in comparison to control group [25]. The same cognitive impairment was also observed in rats that had single 4-h exposure to isoflurane [22]. The cognitive impairment seems to be correlated with persistent decrease in the hippocampal neural stem cell pool and neurogenesis [25].

Intra-peritoneal administration of propofol to seven-day-old rats has also been shown to cause significant cell death in the cortex and hippocampus [26,27]. Administration of propofol (75 mg/kg) once a day for seven consecutive days resulted in more remarkable increase in neuroapoptosis and significant reduction in neuronal density than administration of propofol (75 mg/kg) once only [27]. The researchers also showed that adult rats subjected to repeated administrations of propofol as neonates had significant long-term learning and memory impairment, whereas rats that had only single dose of propofol as neonates did not. The impairment of long-term learning and memory was found to be associated with significantly lower level of the neurotransmitter, glutamate, in the cortex and hippocampus of adult rats [27].

Xenon is well known for its neuroprotective properties in rodents [28]. However, in these studies, xenon was administered at doses between 10% and 75% of an atmosphere, equivalent to a minimum alveolar concentration (MAC) of approximately 0.06–0.79 MAC, based on xenon MAC values of 1.6 atmospheres in rats, and 0.95 atmosphere in mice. To evaluate the effect of xenon on the developing rodent brain is technically difficult as it requires hyperbaric chamber and subjecting young animals to above atmospheric pressure. Nevertheless, a recent *in vitro* study showed that, like isoflurane and sevoflurane, xenon caused neurotoxicity in hippocampal slice cultures from seven-day-old rats when studied at concentrations of MAC and above, and the increase in cell death was not as a result of raised atmospheric pressure [29]. However, whether this study finding can be extrapolated to clinical practice is questionable because the study was performed in an *in vitro* setting and the hyperbaric administration of xenon for anaesthesia is also not practical clinically. Nevertheless, this finding may suggest all anaesthetic agents can potentially result in neurotoxicity. That is to say, there may not be a safe anaesthetic for young animals, but only safe anaesthetic concentration and exposure duration.

In addition to anaesthetics-induced neuronal cell death, changes in the function of surviving neurons caused by anaesthetics in immature brains may also contribute to the development of learning and behavioural deficits later on in life. Recent data indicates that developmental exposure of clinically relevant doses of anaesthetics, such as isoflurane and sevoflurane, cause structural disturbances of developing synapses in hippocampus in both rats and mice [21,22,30], alters dendritic spine density in prefrontal cortex of rats [31,32], reduces expression of key proteins involved in the development of axonal connections [17], and disrupts axon targeting in mouse neocortex [33,34]. These findings suggest that anaesthetic exposure in the early postnatal period might permanently impair circuit assembly in the developing brain. Such disruption in brain circuit development may partly be explained by the recent findings that exposure to anaesthetics, such as propofol and isoflurane, can cause widespread apoptosis of oligodendrocytes in the developing brains of rhesus monkeys. Researchers also showed that oligodendrocytes beginning to acquire myelination competence are especially at risk to the apoptogenic action of anaesthetics [35,36].

Aforementioned studies have shown that commonly used anaesthetic agents increase neuronal cell death if administered during critical periods of brain development. It is ethically not feasible to explore the effects of anaesthetic agents on neuronal cell death in human infants or children. However, the nonhuman primate, including the rhesus monkey, functions as a bridge to decrease the uncertainty in extrapolating rodent data to the human condition. It provides a closely-related animal model appropriate for examining these effects of anaesthetic agents.

Studies have shown recently that exposure to ketamine or propofol for 5 h can cause significant increases in neuronal and glial apoptosis in rhesus monkeys as does a 5 h exposure to isoflurane alone when end tidal concentrations were as high as 1.5% [35,36,37]. Exposure of 70% nitric oxide and 1% isoflurane for 8 h to five- to six-day-old rhesus monkeys has also been shown to cause both neuronal apoptosis and necrosis in the frontal cortex, temporal gyrus, and hippocampus. However, significant neuronal cell death was not observed when either 70% nitric oxide alone or 1.0% isoflurane alone was used. Taking into consideration of the finding that exposure to 1.5% isoflurane alone for 5 h is sufficient to significantly increase neuronal and glial apoptosis [37], 1% isoflurane may require long exposure time to induce neuronal damage or the threshold concentration of isoflurane for inducing neuronal cell death in primates may lie between 1.0% and 1.5%.

With regards to anaesthetics-associated cognitive impairment, recent assessments have demonstrated persistent cognitive deficits in rhesus monkeys exposed to a single 24-h episode of ketamine-induced anaesthesia during the first week of postpartum life [38].

## 3. Mechanisms of Anaesthetics-Induced Neuronal Death

Studies prior to the year 2010 showed many potential mechanisms by which commonly used anaesthetics may induce neuroapoptosis in immature animal brains. Such mechanisms include anaesthetics-induced NMDA receptor inhibition and GABA receptor excitation [39], anaesthetics-induced decrease in neutrotrophic factors [40], and anaesthetics-induced activation of inositol 1,4,5-trisphosphate (IP3) receptors [41]. Among these, anaesthetics-induced NMDA inhibition and GABA excitation continue to be the focus of several studies in the past three years. Beside this, the effects of anaesthetics on mitochondria were another research focus as why immature neurons are subject to anaesthetics-induced apoptosis.

Ketamine is a non-competitive antagonist of NMDA receptors and acts primarily by blocking NMDA ligand-gated channels. A recent *in vitro* study recorded NMDAR channel activity in the form of evoked NMDAR-mediated excitatory postsynaptic currents (eEPSCs) in both immature (post-natal four to seven days) and mature (post-natal three to four weeks) rat forebrain neurons. The authors showed that ketamine produces a greater and longer inhibition on NMDAR channels in immature neurons than in mature neurons. It is plausible to argue that a much more compensatory un-regulation of NMDARs would develop in immature brains following the receptor blockage by ketamine [42]. This argument is supported by recent *in vitro* and *in vivo* findings that exposure to ketamine increased NMDA receptor subtype one (NR1) expression in neurons harvested from the forebrain of new-born rats [43,44,45]. Repetitive exposure to sevoflurane during gestation was also shown to increase NMDA receptor NR1 expression in hippocampus of rat offspring [17]. Such compensatory enhancement of NMDA receptor activity subsequently allows a greater influx of calcium into neurons, leading to an increase in the generation of reactive oxygen species and neuronal cell apoptosis [45]. Co-administration of substances that specifically target the NMDA receptor NR1 subunit has been shown to effectively reverse such neuronal cell death *in vitro* [46].

In contrast to its inhibitory action on adult neurons, GABA acts as an excitatory neurotransmitter in immature neurons [47,48]. Activation of GABA receptors generates action potentials, directly opens voltage-dependent calcium channels (VDCCs), and increases the intracellular calcium concentration in the hippocampus and in other brain structures. This theory is supported by a study that showed isoflurane could increase calcium entry via VDCCs in GABAergic neurons in developing rat hippocampus *in vitro*. As discussed above, an increase in intracellular calcium level is a critical factor in excitotoxic cell damage and can lead to apoptosis. The demise of a significant number of GABAergic neurons immediately after isoflurane exposure has been observed in new-born rats and mice [15,49], and it was associated with a disparate reduction in expression of GABA-synthesizing enzymes [15].

Mitochondrion appears to be the mediator between anaesthetics-induced increase in calcium level and cell apoptosis. Excessive intracellular calcium sequestration can increase reactive oxygen species accumulation inside mitochondria, leading to mitochondrial damage. The injured mitochondria may then release pro-apoptotic proteins, such as BAX and cytochrome *c*, to cytosol, which initiated apoptotic pathway [50,51].

In addition, recent evidence suggests that mitochondria could be subcellular targets of general anaesthesia. When administered during the postnatal period, general anaesthetics cause protracted injury to mitochondria including significant enlargement, disruption of their structural integrity, and a decrease in their distribution in presynaptic neurons [30,52]. Along with morphological changes, general anaesthetics may cause impaired mitochondrial regeneration and function in immature neurons [53]. Indeed, mitochondrial dysfunction is associated with significant up-regulation of reactive oxygen species and the disturbance of neuronal scavenging machinery [53].

Recent study demonstrates that exposure to more than one MAC concentrations of ketamine, isoflurane and nitric oxide for prolonged period (up to 12 h) had no direct cytotoxic effects individually or when given as anaesthetic cocktails on dissociated new-born rodent neurons *in vitro* [54]. However, the studies discussed above showed that significant neuroapoptosis would have been observed should such anaesthetic regimes were used *in vivo* or in cultured brain slices *in vitro*. This discrepancy suggests that neurotoxicity caused by anaesthetics likely requires an intact brain cytoarchitecture or neuroanatomy. Dissociated neuronal cells *in vitro* are not subject to anaesthetics-induced neurotoxicity unless supra-clinical concentrations or prolonged exposures of anaesthetics were given [55].

## 4. A New Model: Human Neurons Differentiated from Embryonic Stem Cells

Human embryonic stem cells (hESCs) are pluripotent stem cells obtained from the inner cell mass of human embryos at pre-implantation stage. hESCs are able to proliferate indefinitely and virtually differentiate into every cell type found in the adult body [56]. Human neural stem cells are cells that differentiate from hESCs and have the ability to replicate, and produce a large number of functional progeny that can differentiate into neurons, astrocytes, or oligodendrocytes [57,58]. They are present throughout development and have been identified in nearly all regions of the embryonic mouse, rat, and human central nervous systems [59,60]. It has been shown that hESC-derived neurons *in vitro* were similar to human neurons at morphological and structural levels [61].

In the recent years, human stem cells-derived models, especially human embryonic neural stem cells, have become a new avenue of research for detecting early-life anaesthetics-induced neurotoxicity and developing potential protection/prevention strategies against such neuronal injury [62]. These cells have provided a potentially invaluable tool for investigating the developmental effects of anaesthetics in human tissues, and yet avoiding the ethical issues around *in vivo* research in human infants and children.

Bai and colleagues, 2013, derived human neural stem cells from hESCs and cultured them for two weeks to obtain differentiated neurons. Neural stem cells or two-week-old differentiated neurons were subsequently incubated with or without 100 μM ketamine for 3, 6, or 24 h [63]. Caspase 3 activity assay showed that 24 h ketamine treatment led to a significant increase in apoptosis in two-week-old neurons but not in neural stem cells, suggesting that neural stem cells are more resistant to cell apoptosis than neurons. As discussed above, such difference in cell susceptibility to ketamine-induced toxicity may be due to the possibility that neural stem cells express fewer NMDA receptors. Moreover, the study showed that 6 h exposure to 100 μM ketamine significantly increased neural stem cell proliferation. The authors argued that such increase in the number of neural stem cells, which was also evident in ethanol-induced early developmental toxicity [64], might contribute to ketamine-induced abnormal brain development [63].

Furthermore, the study showed that significant apoptosis of two-week-old neurons was only observed after 24 h of 100 μM ketamine treatment. However, an earlier study published by the same research group showed that significant decrease in cell viability was observed after 6 h of 3500 μM ketamine treatment [65]. Collectively, these combined data showed that ketamine dose- and time-dependently caused neuronal death. This supports the above statement that there may not be a safe anaesthetic for young people, but only safe anaesthetic concentration and exposure duration. In addition, in both studies, ketamine-induced neuronal apoptosis was accompanied by a significant decrease in mitochondrial membrane potential and an increase in pro-apoptotic protein release from mitochondria into cytosol, suggesting that mitochondria may also play an important role in the process of anaesthetic-induced neuronal apoptosis in humans [63,65].

## 5. Potential Alleviating Strategies for Anaesthetic Neurotoxicity

Since commonly used anaesthetics have shown neurotoxic properties, many studies have searched for potentially mitigating or neuroprotective strategies. A variety of compounds, such as lithium [66], melatonin [67], 7-nitroindazole [68], l-carnitine [69], dexmedetomidine [70], and xenon [51,71], have been previously found to decrease anaesthetic-induced neurodegeneration, although mechanisms by which they work warrant further studies.

In addition to being potential NMDA receptor antagonists and/or GABA receptor agonists, anaesthetic agents can also act directly on a variety of receptors, such as inhibiting metabotropic glutamate receptors (mGluRs). A recent study showed that *N*,*N*′-dibenzhydrylethane-1,2-diamine dihydrochloride (AMN082), an allosteric agonist of mGluR7, could mitigate sevoflurane induced neuroapoptosis in developing rat brains, both *in vitro* and *in vivo* [72]. Administration of AMN082 also significantly improved seveflurane-induced learning and memory defects in rats. The investigators demonstrated that extracellular signal-regulated kinase 1 and 2 (ERK½) signalling pathway was involved in the neuronal protective effects of AMN082 on sevoflurane neurotoxicity [72]. The same research group later on showed that *N*-stearoyl-l-tyrosine (NsTyr), an *N*-arachidonoylethanolamine (AEA) analogue, could also ameliorate sevoflurane neurotoxicity via modulating the ERK½ signalling pathway in neonatal rats [73]. AEA is an endocannabinoid and has been shown to play a neuroprotective role in certain neurodegenerative diseases [74]. Its analogue, NsTyr, could protect neurons from ischemia insult and improve hippocampus dependent learning and memory deficits *in vivo* [75]. Therefore, if AMN082 or NsTyr is proven to be safe for use in paediatric or obstetric medicine, administration of such agents at the induction of anaesthesia might serve as a potential therapeutic approach for mitigating the risk of developmental anaesthetic neurotoxicity.

Moreover, strategies that protect mitochondrial integrity and prevent reactive oxygen species accumulation are also effective in alleviating anaesthetics-induced neurotoxicity.

EUK-134 is a synthetic reactive oxygen species scavenger. R+ pramipexole (PPX) is a synthetic aminobenzothiazol derivative that restores the integrity of mitochondrial membranes. Subcutaneous administration of EUK-134 or intra-peritoneal administration of R+ PPX to post-natal day seven rats around the time of anaesthetic exposure has been shown to significantly reduce reactive oxygen species production, prevent mitochondrial morphological damage, and prevent neuronal loss [76]. Most importantly, researchers also showed that peri-anaesthesia treatment with EUK-134 or R+ PPX prevented anaesthesia-induced learning and memory impairment in adult rats [76,77].

Molecular hydrogen gas is also an effective reactive oxygen species scavenger [78]. Compared to EUK-134 and R+ PPX, hydrogen can be easily supplied as part of the carrier gas mixture under anaesthesia. Yonamine and colleagues showed that co-administration of 3% sevoflurane for 6 h with less than 1.3% hydrogen as part of the carrier gas mixture significantly suppressed neuroapoptosis caused by sevoflurane exposure in six-day-old mice. Concomitant hydrogen inhalation significantly reduced oxidative stress induced by sevoflurane exposure. It mitigated the impairment of long-term memory and the deficit in social interaction behaviours in adulthood caused by neonatal sevoflurane exposure [79].

Beyond the data from rodent studies, prevention of reactive oxygen species accumulation may also decrease anaesthetics-induced neuroapoptosis in humans. Trolox, a water-soluble analogue of vitamin E and a reactive oxygen species scavenger, has been shown to significantly attenuate the increase of ketamine-induced reactive oxygen species production and cell apoptosis in two-week-old neurons derived from human embryonic neural stem cells *in vitro* [63,65].

Apart from targeting the NMDA-mediated neuron excitatory pathway and the reactive oxygen species production, a recent publication showed that co-administration of a non-steroidal anti-inflammatory drug (ketorolac) can ameliorate the sevoflurane-induced cognitive impairment in mice [23].

## 6. Conclusions

All clinically-used general anaesthetic agents are potentially neurotoxic to the developing brain in animals. There may not be a safe anaesthetic for young animals, but only safe anaesthetic concentration and exposure duration. Single exposure to general anaesthetics, during the peak period of synaptogenesis, can result in significant increase of both neuronal and glial cell apoptosis in neonatal animals. The mechanisms of anaesthetics-induced neurotoxicity seem to involve altered expression of ligand-gated ion channels, disturbance to intracellular calcium homeostasis, and mitochondria-mediated apoptotic pathway. In addition, the anaesthetic exposure during the neonatal period is associated with persistent structural and chemical dysfunction of brain cells in adult animals. Such cellular dysfunction may lead to learning and memory (hippocampus-dependent) impairment in adult animals. However, perhaps due to high-grade brain plasticity in young animals, persistent neuronal cell loss was not observed in adult animals, unless repeated and prolonged courses of anaesthesia were administered in neonates. These findings suggest that anaesthetic exposure during the brain growth spurt period may cause persistent functional alterations in remaining neurons and such alternations are not mendable through brain remodelling. Moreover, recent studies suggested that inhibition of excessive NMDA-mediated excitatory pathway, prevention of reactive oxygen species accumulation, and improvement of peri-anaesthesia neuroinflammation can all ameliorate anaesthetics-associated cognitive impairment in animal models. Advances in our understanding of stem cell biology and neuroscience have opened up new avenues of research for investigating early-life anaesthetics-induced neurotoxicity and developing potential prevention strategies against such neuronal injuries. Human embryonic neural stem cells-model might serve as a bridging platform to provide the most expeditious approaches toward decreasing the uncertainty in extrapolating preclinical data to the human condition.
